# Chitosan microsphere-supported catalysts: design, synthesis and optimization for ethylene polymerization†

**DOI:** 10.1039/d4ma00893f

**Published:** 2024-11-28

**Authors:** Joren M. Dorresteijn, Robin Conradi, Laurens D. B. Mandemaker, Kordula Schnabl, Virginie Cirriez, Alexandre Welle, Daniel Curulla-Ferré, Florian Meirer, Eelco T. C. Vogt, Bert M. Weckhuysen

**Affiliations:** a Inorganic Chemistry & Catalysis, Debye Institute for Nanomaterials Science and Institute for Sustainable and Circular Chemistry, Utrecht University 3584 CG Utrecht The Netherlands B.M.Weckhuysen@uu.nl; b R&D Polymers, TotalEnergies One Tech Belgium, Zone Industrielle C Feluy 7181 Belgium

## Abstract

Polyolefins are the main building blocks for consumer products. Here, chitosan, a biopolymer that can be derived from abundant fishery waste, is shaped as a microspheroidal support using spray drying to facilitate ethylene polymerization. Definitive screening design was used to optimize synthesis steps efficiently. The generated catalysts were tested for ethylene polymerization, and the effects of MAO loading and generated porosity were assessed using a variety of micro- and spectroscopic techniques.

## Abstract 2

Chitosan, a biopolymer derived from waste, is shaped as a support to facilitate polymerization. Synthesis optimization was done by definitive screening design, while the generated catalysts were tested for polymerization and analyzed by spectroscopy.

## Graphical abstract

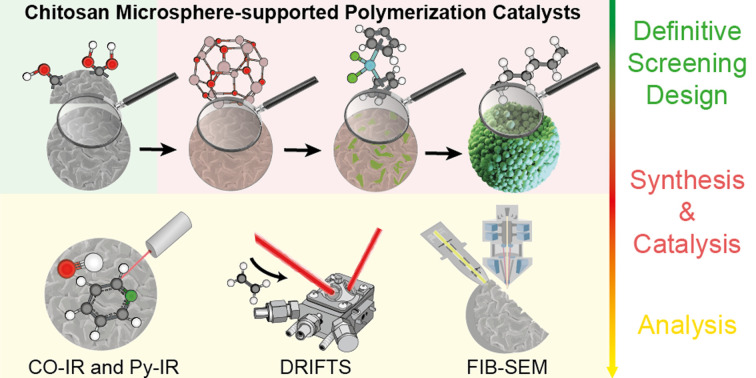

## Introduction

In the global landscape marked by a continuously expanding population, addressing the rising requirements for energy and resources poses a multifaceted challenge. One of the most prominent examples is the production and consumption of synthetic plastics, which have been rapidly growing worldwide, from 2 million metric tons per year in 1950 to 368 million metric tons annually in 2019.^1^ Polyolefins, mostly polyethylene (PE) and polypropylene (PP), are the world's most used plastics covering around 50% of the total plastic production.^2^ Notably, polyolefins follow the twelve green chemistry principles and therefore will play a pivotal role in the energy transition.^3,4^ Also, polyolefin waste will be vital as a carbon source for renewables.^3,4^

PE, among others, is produced using catalysts such as the Ziegler Natta or a metallocene catalyst. Ziegler Natta catalysts comprise a MgCl_2_ support impregnated with TiCl_4_ as the active site, in which alkyl alumina co-catalysts are added to activate the catalyst and produce PE.^5^ The discovery of single-center metallocene catalysts has resulted in significant improvements and advantages over its predecessors in polymerization catalysis and copolymerization of olefins.^6^ An additional benefit is found in these metallocenes as they are recognized as green catalysts and better for the environment than Ziegler-type catalysts.^3^ Metallocenes are usually supported on silica (SiO_2_) gels, produced from hazardous silicon alkoxides by a gelation process.^7^ The silica gels are then impregnated with the co-catalyst methylaluminoxane (MAO). They generally yield better control over polymer stereochemistry, comonomer incorporation, and higher activity. However, they have a faster decay profile, and the catalyst is likely to overheat due to its high initial activity, resulting in fine formation.^8^

Chitosan is a natural linear biopolymer amino saccharide derived from chitin; the second most abundant polysaccharide in the world.^9^ As a waste material, around 100 billion tons of chitin are mainly extracted from the exoskeletons of marine shrimps, lobsters, as well as the cell walls of fungi and yeast. Efforts have been put into utilizing chitin as a feedstock, but the majority is still widely used as landfill, burnt, or disposed into the ocean.^10^ Therefore, utilizing this large waste accumulation as a support material for green polymerization catalysts is a promising route of valorization. Chitin is naturally rather inert, hence it is often converted to chitosan in which a deacetylation of at least 50% has taken place.^9^ Chitosan is soluble in acid and shows great promise in the pharmaceutical industry or as alternative to single-use plastics.^11–14^ It has also been synthesized and tested as support for several heterogeneous metal-supported catalysts in oxidation, hydrogenation, and coupling reactions.^13,15,16^ Finally, relevant to this work, it has been tested as support for olefin polymerization reactions and could have a higher affinity for olefin monomers due to its organic nature, allowing better monomer diffusion inside the catalyst structure and hence could lead to better activity and catalyst fragmentation.^17,18^ Interestingly, chitosan can be synthesized in spherical form, which would be favorable for olefin polymerization reactions as the product will mimic the support due to the replication phenomena.^19^

Additionally, understanding the functional groups of chitosan and their interaction with MAO is essential in further optimizing chitosan as support for metallocene catalysts. The deacetylation of chitin to chitosan yields amino groups, which in theory could have a poisoning effect on the metallocene as it binds irreversibly to these groups.^20^ Fortunately, chitosan also contains hydroxyl groups, which favor the binding of MAO, preventing these poisoning effects.^21,22^

**Fig. 1:**
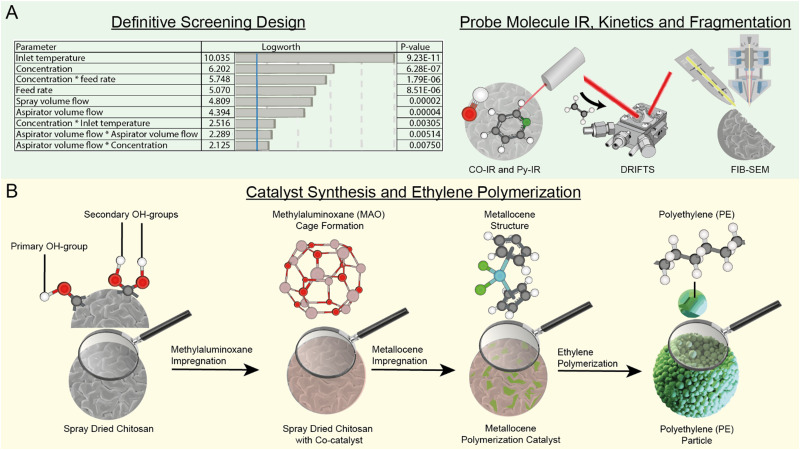
Schematic overview of the methods used in this study. (A) To efficiently optimize the synthesis conditions of spray drying chitosan microspheres, definitive screening design (DSD) was carried out to optimize yield, particle size and outlet temperature (shown here). Subsequently, the synthesized activated supports and corresponding catalysts were analyzed with probe molecule (CO and pyridine) Fourier transform infrared spectroscopy (FT-IR), Diffuse reflectance infrared Fourier transform spectroscopy (DRIFTS) and focused ion beam scanning electron microscopy (FIB-SEM) to study anchoring site formation, kinetics, and fragmentation behavior. (B) Overview of the catalyst synthesis steps and ethylene polymerization with the synthesized catalyst. Chitosan (CS) possesses primary and secondary hydroxyl (OH) groups, which can react with methylaluminoxane (MAO) to produce anchoring sites for the metallocene. The CS/MAO/Zr catalyst is then active for ethylene polymerization for the production of polyethylene (PE).

In this work, we designed and synthesized chitosan microspheres as support in a Zr-based metallocene catalyst. As schematically represented in Fig. 1, we first performed a definitive screening design (DSD) to ensure the preservation of waste in this method by using the least number of experiments that need to be performed to assess the synthesis parameters, as shown in Fig. 1a. How this DSD was performed and what parameters were used is further explained in the ESI† Section S1 and *vide infra*. Then, the resulting catalyst was characterized to understand its Lewis acid sites and potential interaction between the chitosan functional groups and the MAO co-catalyst by using CO- and pyridine probe molecule adsorption IR spectroscopy. Diffuse reflectance infrared Fourier transform spectroscopy (DRIFTS) was used to study the kinetic behavior of the catalyst, additionally after the introduction of pore cavities, which were realized using a hard-templating technique. Finally, focused ion beam scanning electron microscopy (FIB-SEM) was used to image the inside of the microspheres after the polymerization reaction in slurry and gas phase, providing insights into the fragmentation behavior of both the nonporous and porous chitosan-supported metallocene catalyst. Utilizing this toolbox, we designed and followed the different steps of the catalyst optimization within the polymerization process, as represented in Fig. 1b, from the spray drying synthesis to the impregnation with the MAO cocatalyst and the final metallocene catalyst composition leading to the polymer-covered product. This approach demonstrates, on one hand, how DSD and effective catalyst characterization can provide insights on polymerization catalysts in general, and on the other hand how chitosan can be utilized as support for potential future catalyst applications.

## Results and discussion

### Definitive screening design

**Fig. 2:**
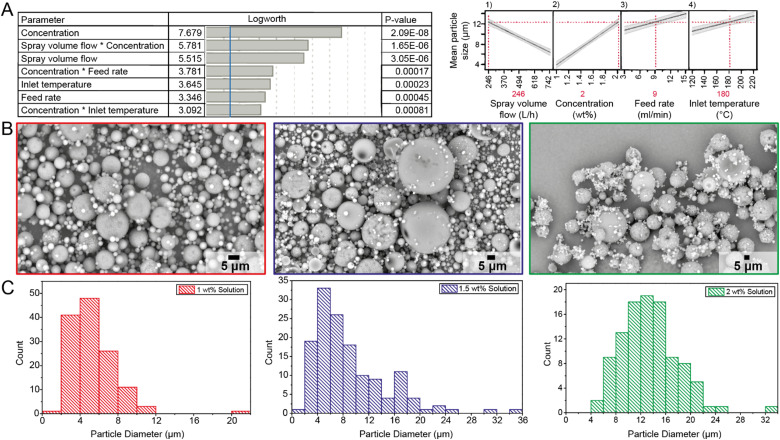
Schematic overview of the definitive screening design (DSD) method used on a key performance indicator (KPI): mean particle size. (A) DSD summary results of performance indicator: Mean particle size. The *p*-value and logworth are indicated for every main effect. The blue line is the threshold *p*-value of 0.05. Right: prediction profiles extracted from the effective model selection for DSDs representing the dependence of the mean particle size on (1) spray volume flow, (2) concentration, (3) feed rate, and (4) inlet temperature. The values for an optimal mean particle size are indicated in red. The shaded areas represent 95% confidence intervals. (B) SEM images of the DSD experiments with different chitosan concentrations, 1 wt% chitosan solution (red), 1.5 wt% chitosan solution (blue), and 2 wt% chitosan solution (green). (C) Particle size distributions (PSD) of DSD experiments with different chitosan concentrations: 1 wt% chitosan solution (red), 1.5 wt% chitosan solution (blue), and 2 wt% chitosan solution (green).

Spray drying is a widely applied drying method to obtain particles with a spherical shape from an aqueous solution or suspension.^23^ In the chemical industry, spray drying is commonly used to synthesize fluid catalytic cracking catalyst particles and large-scale silica supports.^24,25^ Spray drying enables precise control over particle size, morphology, and composition, thus facilitating the uniform distribution of active components within the catalyst structure and enhancing catalytic performance and stability.^26,27^ Since the parameters of the spray dryer significantly conduct the properties of the chitosan, a definite screening design (DSD) is performed to create a model that visualizes every parameter's effect, directing us in using the optimal parameters for the intended chitosan microsphere supports (Fig. 2A).^28^ A more elaborate explanation of the DSD and how the varying parameters would influence the predicted products can be found in ESI† Section S1.

Five parameters (“factors”) were investigated using DSD to optimize yield and particle size to be high enough as a function of different variables (“responses”).^29^ The factors that have the most substantial effect on a response will be identified, and once a parameter is considered to impact a key performance indicator (KPI) significantly, this parameter is denoted as main effect. In this work, the investigated KPIs are outlet temperature, yield, and the microsphere's particle size, which is shown in Fig. 2A as an example. The spray-dried microspheres were analyzed with SEM (Fig. 2B) to determine the size distribution and mean particle size (Fig. 2C). All results are then analyzed using effective model selection for DSD methodology.

Outlet temperature is an important KPI to determine the quality of the spray dry process and the resulting dryness of the spheres. Absorbed water or residual hydroxyl groups could directly deactivate the metallocene, so the residual moisture content at the end of the spray-dry process should be as low as possible.^22^ The DSD summary for this KPI is given in ESI† Fig. S1, and all parameters were considered main effects since they all have a *p*-value less than the threshold *p*-value of 0.05. In addition to these five main effects, four second-order effects are identified, denoted by the combination of 2 main effects. An effect impacts the KPI the most when the coefficient of factor effect is the largest. Since *p*-values can be extremely small when data has a high dependence relationship, taking the logworth (−log(*p*-value)) allows mitigating this effect. The inlet temperature has the largest influence, as also seen in the prediction profiles (Fig. S1, ESI†). Increasing chitosan concentration and aspirator volume increases the outlet temperature, whereas increasing spray volume and feed rate decreases the outlet temperature.

Yield is another KPI, ensuring minimal potential product loss. The yield of the spray-dry experiments is determined by dividing the obtained weight of chitosan in the collection vessel of the spray by the total weight of chitosan in the solution, taking the volume and concentration of the spray-dried solution into account. The model identifies three main effects: spray volume flow, feed rate, and concentration, as highlighted in Fig. S2 (ESI†). Especially the spray volume flow has a significant impact (Fig. S2b, ESI†), as a smaller spray volume flow yields larger droplets. These are more challenging to dry, resulting in larger deposits on the surface of the glass from the drying chamber and finally transported to the collection vessel. As seen in Fig. S2 (ESI†), feed rate and concentration have similar factor effect coefficients, thus impacting the yield with the same magnitude. A higher concentration increases the yield slightly (Fig. S2c, ESI†), and the yield decreases when the feed rate increases (Fig. S2d, ESI†). As quadratic terms for the spray volume flow and concentration are considered second-order effects, the prediction profiles implement this quadratic behavior. According to the model, the yield increases more when the spray volume flow increases from 246 L h^−1^ to 357 L h^−1^ compared to the increase within 357–742 L h^−1^. For concentration, a maximum yield of 24% is obtained at 1.79 wt%, and this decreases further when the chitosan concentration is increased towards 2 wt%.

Finally, the mean particle size of a batch of spray-dried chitosan is essential to control as larger particles from 20 to 80 μm enhance the flowability and the catalyst feed injection within an industrial polymerization process.^30^ However, using our lab-scale spray dryer, consistently obtaining particles larger than 20 μm is a challenge. An effective model selection is performed to analyze which parameters impact the mean particle size the most. According to the DSD summary results shown in Fig. 2a, the concentration, spray volume flow, feed rate, and inlet temperature have a significant impact on the particle size, in which concentration and spray volume flow play the biggest role. The mean particle size increases if the chitosan concentration increases, as is seen in Fig. 2B-blue, and confirmed by the SEM images (Fig. 2B) and corresponding particle size distributions (Fig. 2C) of chitosan microspheres synthesized using 1, 1.5, or 2 wt%, respectively. For these concentrations, the mean particle size increased from 5.29 μm to 9.10 μm to 13.48 μm, and the distribution broadened as well. The inhomogeneous particle size could be explained by the nozzle of our spray dryer not producing uniform droplets. When spray drying takes longer, particle deposits can build up at the nozzle tip and decrease the nozzle's diameter, hence resulting in smaller droplets afterwards. This effect is pronounced as the concentration increases; at 2 wt%, the solution also seemed too viscous for efficient spray-drying, making atomization more challenging and creating a larger variety of droplet sizes. Still, the higher concentration also yielded a larger mean particle size, which was intended.

Bigger droplets are produced when the spray volume flow decreases to 246 L h^−1^ and the feed rate increases to 15 ml min^−1^. These bigger droplets will result in a larger mean particle size after drying. When the inlet temperature increases, shell formation inside the droplets will happen very early in the spray-dry process. A higher inlet temperature can lead to larger but hollow particles due to diffusion limitation during spray drying, which is unfavorable for a polymerization catalyst.

The optimal parameters for a maximized mean particle size are in direct contrast to the parameters for a maximized yield. Therefore, a compromise between yield and mean particle size was made to produce a larger batch of chitosan microspheres for testing as catalyst support in the ethylene polymerization reaction. Since a spray volume flow of 742 L h^−1^ ensures optimal atomization, this high spray volume flow was always chosen for a large-batch production, even when considering the effect of a reduction in mean particle size. The microspheres produced as supports for the polymerization reaction, hence for the remainder of this work, are generated setting 37.7 m^3^ h^−1^, 742 L h^−1^, 3 ml min^−1^, and 180 °C for the aspirator volume flow, spray volume flow, feed rate, and inlet temperature, respectively. This procedure yields chitosan microspheres of around 6.82 μm, a final yield of 44%, and an outlet temperature of 118 °C, which is shown in the ESI.† The prediction profiles of the KPI's can be found in the ESI.†

### MAO activation and characterization

After assessment of the parameter optimization for the spray drying procedure, optimized chitosan microspheres were activated with different amounts of MAO loadings and subsequently impregnated with a metallocene (Cp_2_ZrMe_2_) to yield active catalysts for ethylene polymerization (Table 1).

**Table 1:** Elemental Composition of the MAO-impregnated chitosan and corresponding catalysts as determined with ICP-OES (Al wt%, Zr wt%), calculated Al/Zr molar ratio with corresponding activity in ethylene polymerization

(Catalyst)/activator	Al (wt%)	Zr (wt%)	Al/Zr ratio	Activity^b^ (gPE/gCat × h)
(Zr)/CS-0Al	0	—	—	—
(Zr)/CS-23Al	22.76	(2.5)^a^	9.10	6.94
(Zr)/CS-30Al	29.54	(2.5)^a^	11.8	11.40
(Zr)/CS-36Al	36.79	2.21	16.6	13.76
(Zr)/CS-46Al	46.31	2.57	18.0	20.82

a Expected Zr loading, because these samples could not be measured with ICP-OES.

b Polymerization conditions: room temperature, heptane slurry, 1.98 mg mL^−1^ co-catalyst TiBA, 1.2 bar ethylene pressure for 1 hour with 18 mg catalyst.

**Fig. 3:**
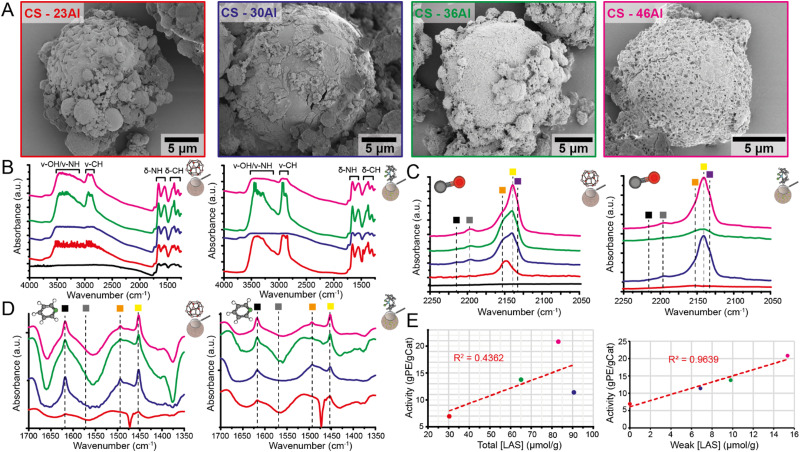
Overview of the scanning electron microscopy (SEM), (probe molecule) IR spectroscopy and ethylene polymerization activity correlations performed on the activated chitosan (CS) microspheres and corresponding catalysts. (A) SEM images of the chitosan microspheres with increasing MAO loading (red) 23% Al, (blue) 30% Al, (green) 36% Al, and (pink) 46% Al. (B) FT-IR spectra of the untreated chitosan support (black) and MAO impregnated chitosan supports CS-(0–46%) Al and respective catalysts Zr/CS(23–46) Al recorded in vacuum at room temperature. (C) FT-IR spectra for the untreated chitosan support (black) and MAO-impregnated chitosan CS-(0–46) Al and (b) corresponding catalysts Zr/CS-(23–46) after CO adsorption at 85 K at 1 bar. The indicated bands correspond to CO adsorbed on moderate LAS at 2212 cm^−1^ (black), weak LAS at 2198 cm^−1^ (grey), hydroxyl groups at 2151 cm^−1^ (orange), O^2−^ at 2140 cm^−1^ (yellow), and physisorbed CO in pores at 2132 cm (purple). (D) FT-IR spectra of the MAO-impregnated chitosan supports CS-(23–46) Al and corresponding catalysts Zr/CS-(23–46) Al after pyridine adsorption and subsequent temperature programmed desorption (TPD) treatment at 423 K in vacuum. The indicated bands correspond to pyridine adsorbed on Lewis acid sites: at 1618 cm^−1^ (black), at 1574 cm^−1^ (grey), at 1494 cm^−1^ (orange), and at 1452 cm^−1^ (yellow). (E) Ethylene polymerization activity of the catalyst plotted *versus* the concentration of total LAS as determined with pyridine FR-IR spectroscopy and *versus* the concentration of weak LAS as determined with CO FT-IR spectroscopy in the corresponding MAO-impregnated chitosan samples. Trendlines are indicated with a red dotted line with the respective *R*^2^ displayed. All FT-IR spectra are normalized for sample density and plotted with an offset for clarity.

SEM was employed to visualize the grafting of MAO onto the chitosan microspheres (Fig. 3A). When a relatively low MAO content is used (Fig. 3A red–blue), the MAO will deposit only in the wrinkles and between the larger and smaller microspheres. This insufficient MAO loading causes an inhomogeneous distribution of MAO on the surface, which leads to an incomplete coverage of these microspheres. Not all surface hydroxyl groups have reacted with MAO in these cases, which can lead to catalyst deactivation. A higher MAO loading will lead to a more complete coverage of the surface (Fig. 3A-green), which becomes more evident when the MAO loading increases even further to 46% Al loading (Fig. 3A-pink). At this highest loading, the impregnation of MAO also includes porosity on the surface of the microspheres. All particles retain their original shape after MAO impregnation, although MAO acts as a glue to bind these together.

FT-IR spectroscopy was employed to study the vibrational properties of the MAO-impregnated chitosan and the corresponding catalysts. Fig. 3B displays the FT-IR spectra at room temperature under vacuum for the bare chitosan (Fig. 3B-black) and the MAO-impregnated chitosan (Fig. 3B-colored). In the IR spectrum of the chitosan support without MAO, a broad band appears from 3700 to 3000 cm^−1^, corresponding to the stretching vibrations of both the hydroxyl and amino groups of chitosan.^31^ Moreover, chitosan does not contain isolated surface hydroxyl groups, like in silica.^22^ These hydroxyl groups are all orientated differently in chitosan and display this extensive band. At low wavenumbers, the bands between 1660 and 1500 cm^−1^ are attributed to the bending vibration of the amino groups. The bands between 1450 and 1250 cm^−1^ are attributed to the CH bending vibrations.^31^ When MAO is impregnated on the chitosan support, bands between 3100 and 2800 cm^−1^ appear, arising from the CH stretching vibrations of methyl groups of MAO.^22^ The band of the hydroxyl vibrations is partly perturbed due to MAO reacting with these hydroxyl groups.^22^ MAO can only react to the chitosan on the surface and not inside the microspheres. Since FT-IR is a bulk spectroscopy technique, all vibrations in the microspheres are measured, which results in this broad, perturbed hydroxyl band. Fig. 3B-right shows the IR spectra of the supported metallocene catalysts. The δ-CH and ν-CH bands do not alter significantly when the activated chitosan is impregnated with the metallocene precursor. Typically, the cyclopentadienyl ligands will produce extra vibrations in the δ-CH bands around 1508, 1470, 1445, and 1384 cm^−1^.^32^ However, these are not clearly visible.

The utilization of FT-IR spectroscopy in combination with two probe molecules, pyridine and CO, allows the examination of the acidic properties of the MAO-impregnated supports and the corresponding catalysts. Pyridine is a harder Lewis base than CO, and can therefore provide a reliable indication of the overall Lewis acidity of the samples.^33^ CO, a softer Lewis base, reacts more sensitively to local coordination states, therefore offering more detailed insights into the strength of different Lewis acid sites in the samples.^22^Fig. 3C displays the resulting spectra for the MAO-impregnated chitosan (left) and corresponding catalyst (right). At lower wavenumbers, the interaction of CO with hydroxyl groups of the chitosan support is characterized by the band of 2151 cm^−1^.^34^ It is, therefore, surprising that the unimpregnated chitosan does not show any CO adsorption. It would be expected that CO is at least physisorbed in the pores and cracks of the microspheres. It could be that the internal hydroxyl groups of chitosan interact too strongly with each other, preventing CO absorption. When the MAO loading increases, two additional bands at 2140 and 2132 cm^−1^ appear. The band at 2140 cm^−1^ is designated for the CO adsorption to O^2−^ species of MAO.^35^ More MAO deposits on the chitosan microsphere surface are created when the MAO concentration increases, to which CO can adsorb. Furthermore, the band at 2132 cm^−1^ is designated to physisorbed CO in pores.^35^ At higher loadings, MAO can introduce porosity on the surface of the chitosan microspheres (Fig. 3A-pink). At higher wavenumbers, two CO vibrational bands appear at 2212 and 2198 cm^−1^ for the higher loaded MAO samples (Fig. 3B blue–green–pink). These bands indicate the presence of moderate Lewis acid sites (M-LAS) and weak Lewis acid sites (W-LAS), respectively, whose intensity increases when the MAO loading increases.^34^ Weak LAS comprises an aluminum atom bounded by an oxygen atom and two methyl groups (AlOMe_2_). In moderate LAS, this aluminum atom is bounded by two oxygen atoms and one methyl group (AlO_2_Me). This aluminum center is, therefore, a stronger acid because of the electron-withdrawing inductive effect of the extra oxygen atom.^36^ Moderate LAS are less prominent in the MAO-impregnated chitosan samples. The study of Velthoen *et al.* also shows this same trend in CO adsorption for MAO-impregnated silica for traditional metallocene olefin polymerization catalysts.^22^ However, the wavenumbers for CO adsorbed on OH and O^2−^ and physisorbed CO of the MAO-impregnated chitosan are all shifted to slightly lower wavenumbers, indicating a weaker interaction between CO and these groups. The weak and moderate LAS in the chitosan samples have the same vibration as for the MAO-impregnated silica.

Fig. 3B-right displays the CO-IR spectra of the catalysts. The catalysts with 30 and 46 wt% Al (blue and pink) show a similar CO adsorption in the IR spectra as the MAO-impregnated samples. Therefore, the nature of the Lewis acid sites does not change when the metallocene is impregnated to the support in these samples. These spectra also indicate the presence of both weak and moderate LAS, to a lesser extent than the corresponding MAO-impregnated samples. These LAS are, however, not detected in the catalysts with 23 and 36 wt% Al (red and green) because these spectra have a high absorbance in the CO adsorption region. Therefore, these sites are difficult to quantify but could still be present at low, undetectable concentrations.

Quantification of total LAS concentration was done by pyridine FT-IR. Fig. 3D depicts the normalized spectra of the MAO-impregnated chitosan (left) and the corresponding catalysts (right) in the C–H region after the temperature treatment. Pyridine can probe one kind of Lewis acid site, indicated by the four bands that appear at 1618, 1574, 1494, and 1452 cm^−1^. These bands correspond to the ring vibrations of the pyridine molecule when it is chemisorbed to a Lewis acid site. Pyridine also distinguishes between the Lewis acid sites, originating from Al^3+^ with an octahedral (1614 cm^−1^) or tetrahedral (1622 cm^−1^) coordination. The octahedral Al^3+^ are weaker acid sites than the tetrahedral Al^3+^.^37^ Since the pyridine vibration of the MAO-impregnated chitosan is centered around 1618 cm^−1^, both these Al^3+^ coordination sites are present in the samples.

The activity of the catalyst during polymerization in gPE/gCat depends on the total LAS and weak LAS concentration in the catalyst. Fig. 3E shows the correlations between the total LAS concentration (left) in the activators as determined with pyridine FT-IR and the polymerization activity of the corresponding catalysts. The weak LAS concentration is especially linearly correlated with the polymerization activity (right), as shown in Fig. 3E as determined with CO-IR. A higher MAO loading increases the weak LAS concentration, leading to increased ethylene polymerization activity. The immediate interaction between the AlMe^2+^ species of the weak LAS and the metallocene precursor is essential for the activation process.^22^ Purposely, the LAS concentration of the activators, rather than the catalysts, is correlated with the polymerization activity. The interaction between the metallocene precursor and the LAS in the MAO-impregnated chitosan is essential in the activation process. This specific interaction determines the quantity of the monomethylated metallocene species, which are the active catalysts for olefin polymerization.^38^ The LAS concentration in the chitosan samples is lower than the reported silica-supported metallocenes.^22,39^ Nevertheless, this increasing trend from silica-supported metallocenes is also present in the chitosan-supported metallocenes. An MAO loading of at least 30 wt% is necessary to provide enough weak LAS and quench the remaining hydroxyl groups of the chitosan support. In the case of silica-supported metallocenes, a much lower MAO loading of 14 wt% is required to obtain an even higher LAS concentration and polymerization activity than the chitosan-supported catalysts.^22^ Also, the high hydroxyl concentration of chitosan requires a large quantity of TMA species in MAO to quench these groups. Therefore, less weak LAS are left to activate the metallocene precursor.

Another essential aspect of metallocene activation in silica-supported catalysts is the formation of counter anions that stabilize the cationic active metallocene and should not be too tightly bound.^40^ Otherwise, the steric hindrance of a strong LAS inhibits the monomer insertion during polymerization, which yields an inactive catalyst species. The presence of weak LAS in the MAO-impregnated chitosan suggests that this loose ion pair is also present during polymerization, enhancing catalyst activity.

### Catalytic testing

**Fig. 4:**
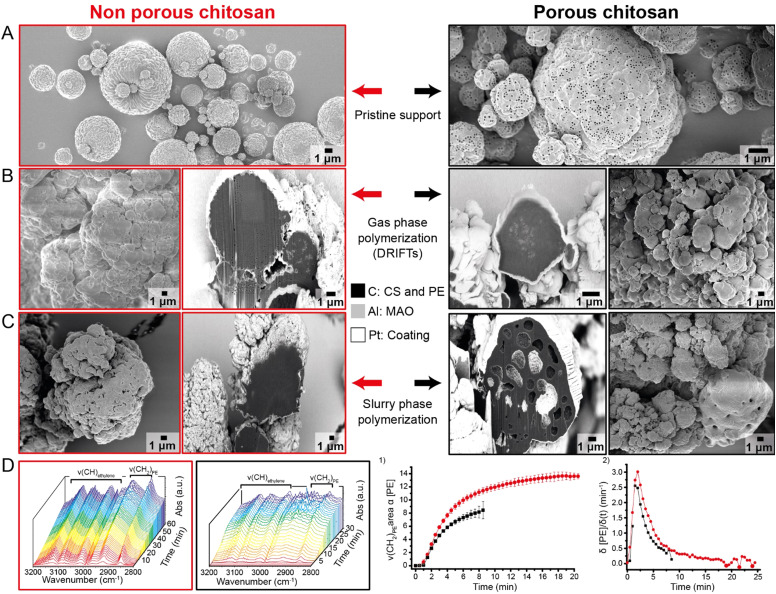
Overview of the analysis done on the nonporous (red) and porous (black) chitosan catalysts. (A) Scanning electron microscopy (SEM) images of nonporous chitosan and porous chitosan microspheres prepared by (polystyrene templated) spray drying. (B) Polymer morphology evaluation of nonporous and porous catalyst during gas phase polymerization and subsequent fragmentation behavior visualized by focused ion beam scanning electron microscopy (FIB-SEM). (C) Polymer morphology evaluation of nonporous and porous catalyst during slurry phase polymerization and subsequent fragmentation behavior visualized by FIB-SEM. (D) Ethylene polymerization kinetics probed with *in situ* diffuse reflectance infrared Fourier transform (DRIFT) spectroscopy. Evolution over time of the DRIFT spectra of the (red) nonporous and (black) porous catalyst while flowing ethylene at a rate of 2.5 ml min^−1^. The polymer expansion during ethylene polymerization causes an increase in absorbance. The FT-IR polyethylene stretching vibration ν(CH_2_) area at 2852 cm^−1^ of the growing PE polymer chains is proportional to the polyethylene concentration [PE] over time. The standard deviation from the average curve, derived from three experiments with the same catalyst sample, is reported by the vertical bars. (1) The first derivative of the curves in (2) reflects the polymerization rate (*δ*[PE]/*δt*) over time.

The chitosan microspheres are tested as catalyst for the polymerization reaction within both very mild slurry and gas phase reaction conditions. However, as the spheres themselves do not show any nano- or microporosity, it is expected that the polymer only forms on the outer shell of the spheres and fragmentation will not occur; however, this would be favorable for the activity and yield of the catalyst as it would expose fresh active sites on the surface of the fragments.^41^ To introduce additional porosity, hard-templating using PS microbeads of 150 nm in diameter was done as the low solubility of chitosan within organic solvents allows the etching of these spheres after spray-drying synthesis using toluene. Fig. S3 (ESI†) shows SEM images of chitosan microspheres with 5 : 1 and 3 : 1 CS : PS ratios after etching, revealing spherical-shaped cavities or pores visible on the surface. A lower CS to PS ratio results in more pores, as is expected. It is however likely that the spheres did not form a fully interconnected pore network using these concentrations, as the spray-drying procedure prevents the synthesis of a “cake” of oriented PS beads as was reported for *e.g.* metal–organic frameworks.^42^ Hence, we proceeded with the 3 : 1 chitosan : PS ratio for catalytic testing, compared to a non porous chitosan microsphere, as shown in Fig. 4A. The catalysts were tested in both gas (Fig. 4B) and slurry (Fig. 4C) phase polymerization of ethylene. (FIB-)SEM was used to study the morphology of the product and catalyst after the reaction, and DRIFT spectroscopy (Fig. 4D) was used to probe the kinetics of the reaction within the gas phase only for both catalysts. Fragmentation in polymerization catalysis is an essential process that must happen during polymerization. To better understand if fragmentation occurs in the chitosan-supported catalysts, FIB-SEM is used to visualize the interior morphology of the polymerized catalyst particles. However, as both the chitosan support and PE are based on carbon, visualizing the fragmentation behavior by FIB-SEM can be challenging.

The nonporous and porous catalysts do contain a significantly high loading of the MAO co-catalyst, based on aluminum (Al) with a higher atomic number. This co-catalyst is bound at the surface of the chitosan microspheres to form an Al/C composite locally. Consequently, the fragmentation pattern of the catalyst could still be visualized by looking at the distribution of Al inside the polymerized PE. Fig. 4B-red depicts the cross-section of a PE particle polymerized by the nonporous catalyst. Here, fragmentation only occurs at selected particles with a small particle size.^19^ These tiny particles are likely to break bisectionally, causing the alumina to be distributed more homogeneously throughout the PE particle. This homogeneous alumina distribution is also further characterized in the EDX-elemental map of this cross-section, displayed in Fig. S4 (ESI†). In the larger PE particles, Al is only present at the sphere's surface. Catalyst particles with a large particle size are likely to fragment in a layer-by-layer fashion. Polymerization occurs from the outside, causing the catalysts to be pulled apart, creating hollow spheres in the polymer particle. However, this hollow sphere polymer morphology is not desirable for the current application.

The Al co-catalyst fragments were not found in the PE particles that reacted during slurry polymerization (Fig. 4C). The ethylene monomer concentration in the slurry is often much lower than that during gas-phase polymerization. During the slurry process, polymerization occurs in much milder conditions, which likely causes less stress build-up inside the catalyst particles.^43^ Polymerization occurs more gradually and less rigorously. Therefore, the large Al co-catalyst fragments are not visible in the FIB-SEM images. However, fragmentation occurs much more gradually throughout the catalyst during slurry polymerization, which is challenging to observe with this technique.

The porous catalyst in Fig. 4B-black has a similar fragmentation pattern as the nonporous catalyst, suggesting that the limited extra porosity of the PS spheres did not significantly influence the catalyst's fragmentation. A significant change in internal polymer morphology occurs for the porous catalyst at milder slurry polymerization conditions. An extensive pore network is created inside the PE particle. This can be explained by the fact that the porous catalyst still contains the PS nanosphere template deeper inside the sphere, as non-surface PS spheres were likely protected by CS during the removal of the template. The spaces these nanospheres occupy in the catalyst can be considered inert zones.^44^ It is assumed that the agglomerates of nanospheres create pores in the growing polymer due to the replication phenomenon. A similar procedure is used in the Ziegler–Natta catalyst to increase the rubber content during high-impact polypropylene (hiPP) production by implementing inactive silica sites in the framework.^44^ This more extensive pore space in the pre-polymerized catalyst is required to prevent sticking during rubber incorporation. Furthermore, the macropores induced after the chemical etching of the PS nanospheres in the chitosan support will cause additional porosity in the final polymer product. In this context, the polymerized porous chitosan catalysts look remarkably like this hiPP morphology. Therefore, this catalyst could have potential in the production of hiPP when overcoming the extensive hydroxyl group content challenge.

At last, the porous and nonporous catalysts show very similar kinetic behavior in the initial reaction stage in Fig. 4D, which was measured by DRIFT spectroscopy under very mild gas-phase conditions. The rate of polyethylene formation was followed by measuring infrared spectra at specific short time intervals while ethylene gas flowed through the reaction chamber. The free rotational characteristic bands between 3250–2900 cm^−1^ were immediately visible in the IR spectrum, shown in Fig. 4D in the second red spectra. Then, two additional bands appeared, centered around 2852 cm^−1^ and 2928 cm^−1^, originating from the symmetrical ν_s_(CH_2_) and asymmetrical ν_as_(CH_2_) stretching vibrations of polyethylene, respectively. Fig. 4D-red shows the nonporous catalyst's spectra evolution for 60 minutes of polymerization time. Due to the expansion of the polymer during the DRIFTs measurements, a larger quantity of the incoming IR laser is absorbed into the polymer particles. Consequently, the intensity of the reflected signal is decreased, which causes the entire spectrum to increase to higher absorbance. This higher absorbance resulted in more noise in the spectrum. Therefore, the PE concentration could only be measured during the initial polymerization phases. This increase in absorbance was especially evident when polymerization was performed with the porous catalyst, as shown in Fig. 4D-black. Oversaturation occurs here after 15 minutes of polymerization, where the nonporous catalyst could be monitored for over 40 minutes before the signal becomes too noisy.

The early-stage kinetics curves of both catalysts were obtained by plotting the area of the polyethylene band at 2852 cm^−1^ over time, revealing the change in PE concentration during polymerization (Fig. 4D-1). The polymerization rates in Fig. 4D-2 were obtained by taking the derivative of the curves in Fig. 4D-1 to compare the relative rates of both catalysts. The initial polymerization nonporous catalyst, peaking at 3 min^−1^, was higher than the porous catalyst, peaking at 2.5 min^−1^. After this initial acceleration, polymerization continued at a steady state. The expansion of the catalyst bed due to the formation of PE caused more noise in the IR spectrum in the later stages of polymerization. At this point, analysis of the DRIFT spectra was no longer valid to obtain relevant kinetic data. The data points obtained after IR oversaturation were inconsistent and are therefore excluded from the figure. It must be noted that this IR oversaturation occurred earlier for the porous catalyst, which is depicted by the enlargement of the standard deviation for every data point after 5 minutes of polymerization time. IR oversaturation and particle expansion for the nonporous catalyst were less notable and occurred much later in the polymerization reaction. The active sites of the nonporous catalyst are all situated at the surface of the support material. A thick PE shell quickly forms around the catalyst particle, preventing further monomer incorporation into the polymer chains.^45^ This diffusion limitation is characterized by the drop in activity after the initial acceleration in Fig. 4D-2. This same trend is visible for the porous catalyst, although it occurs much quicker. Therefore, another phenomenon must cause this faster polymer expansion. It was hypothesized that introducing porosity should lead to quicker fragmentation, which likely occurs during or after IR oversaturation.

The kinetic behavior of chitosan-supported metallocenes is similar to that of the silica-supported metallocenes in the initial phase.^46^ After the decrease in activity, a sudden increase in activity should occur due to the fragmentation of the catalyst by exposing fresh active sites; however, this phenomenon is not observed due to oversaturation. When the thin polymer layer is formed around the catalyst, polymer growth occurs from the outside to the inside, accompanied by a slowly beginning layer-by-layer fragmentation of the support.^45^ This second rise in activity does occur for silica-supported metallocenes but was not observed in the DRIFTS data for both catalysts. However, from the FIB-SEM measurements on both polymer samples, it is clear that both catalysts do fragment after a certain time. Therefore, the fragmentation seems to be milder and less influential on the polymer formation rate than for silica at the initial reaction stage. In the end, the final activity of all produced catalysts were low, which are caused by the very mild ethylene polymerization conditions and too high Zr content in the range of 2.2–2.7 wt%, which ideally would be lower between 0.2–0.3 wt% Zr content. Furthermore, more deacetylation of the chitosan as shown in a previous study could also further improve the activity to higher polymerization activity.^17^

## Conclusions

An exploratory multi-step approach has been utilized to investigate the potential of shaping chitin waste into microspheroidal supports for metallocene-based ethylene polymerization. A definitive screening design was performed to optimize the production parameters and investigate which parameters significantly impact the key performance indicators. A compromise between large particle sizes and high yields was necessary, and optimal parameters were identified for different synthesis goals.

A series of chitosan-supported activators and corresponding catalysts with increasing MAO loading and constant metallocene loading were prepared. SEM revealed MAO distribution on the microsphere's surface. A higher MAO loading leads to more homogeneous coverage and larger particles, acting as a glue between microspheres. The utilization of FT-IR spectroscopy combined with pyridine and CO probe molecule absorption allowed for the examination of the Lewis acidic properties of the MAO-impregnated supports and the corresponding catalysts. The minimal MAO loading of 30 wt% was required to quench all chitosan hydroxyl groups and provide weak LAS for efficient metallocene activation. Higher alumina content increases total and weak LAS concentrations, enhancing polymerization activity. An MAO loading of 46 wt% was recommended to maximize the polymerization activity. Chitosan therefore displayed a similar trend as silica in terms of anchoring properties. However, the high amount of hydroxyl group content indicates a very significant difference in terms of MAO required for polymerization activity and avoiding hydroxyl poisoning.

In addition to the nonporous support, a porous chitosan support was successfully synthesized with template-assisted spray drying using PS nanospheres. Larger PS nanosphere size increased the pore size, but achieving a fully extended pore structure was challenging. We therefore suggest to experiment with other hard-templates such as rods or fibers, as they might increase the odds of generating an interconnected pore network.^47,48^*In situ* DRIFT spectroscopy revealed the early-stage polymerization kinetics, showcasing a high initial activity followed by a steady state rate. The fragmentation behavior could not be visualized with DRIFTs because of an oversaturated signal by the fast polymer expansion of the catalyst.

Fragmentation of the catalyst was visualized using FIB-SEM and only visible after gas-phase polymerization for both nonporous and porous catalysts. We revealed distinct fragmentation patterns, with smaller particles exhibiting bisectional fragmentation and larger particles undergoing layer-by-layer fragmentation. Under slurry conditions, the porous catalyst exhibited a porous PE structure, showing potential for hiPP production. Conclusively, this work demonstrates how designing strategies can lead to optimized syntheses and minimalize experimental waste, here applied to chitosan microspheres within a spray-drying synthesis setup. Subsequently, the microspheres showed promising behavior as green alternative support materials for metallocene-catalyzed polymerization reactions, valorizing one of the most abundant sources of bio-waste as a main catalytic component.

## Experimental

### Spray drying of chitosan microspheres for the definitive screening design

The chitosan microspheres were prepared with a 1, 1.5, and 2 wt% chitosan (98% purity, ≥75% deacetylated, Sigma-Aldrich) solution in 1 wt% acetic acid (≥99% glacial, ReagentPlus®, Sigma-Aldrich)/water solution. The chitosan was dissolved for 24 hours under stirring at room temperature. About 50–100 ml of the solution was used for each experiment and was spray dried using a BÜCHI B-290 acid-resistant mini spray dryer (Flawil, Switzerland) according to the conditions in Table S2.1 (ESI†). This design of experiments was created by implementing the lower and upper limits of all five continuous factors in the program JMP. The program adds four extra runs to identify any second-order effects. During spray drying, the solution was magnetically stirred at 500 rpm to prevent concentration gradients. The outlet temperature was measured, the dry and white chitosan powder was collected in the collection vessel, and the yield was determined. Additional nozzle cooling was provided by feeding pressurized air into the nozzle to prevent precipitation on the nozzle tip. The standard 1.4 mm in diameter spray nozzle tip and 2.2 mm nozzle cap were used for all experiments.

### Preparation of chitosan microspheres

The chitosan microspheres for the catalyst synthesis were prepared with a 1.5 wt% chitosan (98% purity, ≥75% deacetylated, Sigma-Aldrich) solution in 1 wt% acetic acid (≥99% glacial, ReagentPlus®, Sigma-Aldrich)/water solution. The chitosan was dissolved for 24 hours under stirring at room temperature. Spray drying was performed using the BÜCHI B-290 acid-resistant mini spray dryer (Flawil, Switzerland) equipped with the standard 1.4 mm in diameter spray nozzle tip and 2.2 mm nozzle cap. The aspirator volume flow, spray volume flow, feed rate, and inlet temperature were set at 37.7 m^3^ h^−1^, 742 L h^−1^, 3 ml min^−1^, and 180 °C, respectively. These parameters for an optimized yield were acquired by the DSD analysis in the previous chapter. Additional nozzle cooling was provided by feeding pressurized air into the nozzle to prevent precipitation on the nozzle tip. After spray drying, the yielded chitosan powder was further dried in an oven at 120 °C for 24 hours before storing it inside the glovebox.

### Supported activator synthesis

All further steps in this procedure were conducted under an N_2_ atmosphere inside a glovebox. All solvents used for the syntheses are of anhydrous grade and dried over 3 Å molecular sieves. Chitosan was impregnated with an increasing MAO content. In such synthesis^14^, 3 ml, 4 ml, 5 ml, or 6 ml of MAO solution (precursor: Albemarle 30% MAO solution) was dropwise added to a chitosan/toluene slurry in a round-bottom flask containing 0.5 gram of spray-dried chitosan in 20 ml toluene (Fischer Chemical, purity 99.85%). Subsequently, the mixture was heated above toluene reflux temperature (*ca.* 398 K) under gentle agitation for 4 hours at 110 rpm. The suspension was filtrated, and the residue was washed three times with toluene and pentane (Fischer Chemical, purity 99.5%) to obtain weight loadings of 22.76%, 29.54%, 36.79%, and 46.31% (CTS-(23–46)Al). In this notation, the number preceding the Al indicates the weight percentage in a sample determined with inductively coupled plasma optical emission spectrometry (ICP-OES), displayed in Table 1.

### Catalyst synthesis

The supported catalyst of each activator was synthesized by dissolving the chitosan/MAO activator into a toluene solution in a round-bottom flask containing a determined quantity of bis(cyclopentadienyl)dimethyl zirconium(iv) to reach a targeted zirconium content of 2.5 wt%, determined with ICP-OES. After magnetically stirring at 110 rpm for two hours at room temperature, the red-colored slurry was filtered and washed once with toluene and pentane to yield the supported catalysts (Zr-CTS(23–46)Al).

### Synthesis of porous chitosan microspheres using polystyrene nanospheres

The procedure for producing porous chitosan microsphere (PCMS) support consists of two steps. In the first step, the spray drying technique was used to prepare microspheres comprised of the host agent chitosan (CTS) with the template agent polystyrene (PS) nanospheres. Afterward, the PS template is removed by chemical etching with toluene to dissolve PS.

The CTS/PS microspheres were made with a 1.5 wt% chitosan (98% purity, ≥75% deacetylated, Sigma-Aldrich) solution in 1 wt% acetic acid (≥99% glacial, ReagentPlus®, Sigma-Aldrich)/water solution. The chitosan was dissolved for 24 hours under stirring at room temperature. Then, a 1 wt% suspension of polystyrene nanospheres of 150 nm ± 3 nm in size (Nanosphere^TM^ Size Standards, Thermo Scientific) was dropwise added under stirring. The mass ratio of CTS to PS was set to 5 : 1 and 3 : 1. The obtained suspensions were sonicated for five minutes in an ultrasonic bath before spray drying to obtain a homogeneous distribution of the PS nanospheres.

Spray drying was performed using the BÜCHI B-290 acid-resistant mini spray dryer (Flawil, Switzerland) equipped with the standard 1.4 mm in diameter spray nozzle tip and 2.2 mm nozzle cap. The aspirator volume flow, spray volume flow, feed rate, and inlet temperature were set at 37.7 m^3^ h^−1^, 742 L h^−1^, 3 ml min^−1^, and 180 °C, respectively. The DSD analysis acquired these parameters for an optimized yield in Chapter 2. Additional nozzle cooling was provided by feeding pressurized air into the nozzle to prevent precipitation on the nozzle tip.

The spray-dried CTS/PS particles were thoroughly washed with toluene five times to dissolve the PS template agent in the chemical etching process. After each washing step, the microspheres were centrifuged at 5000 rpm for five minutes. Then, the etched particles were washed with ethanol and dried for 24 hours at 120 °C to yield the completely dried and solvent-free PCMS stored in the N_2_ glovebox.

### Slurry ethylene polymerization

The synthesized catalysts were tested for ethylene polymerization at room temperature in a slurry-phase glass reactor filled with 15 mL 99+% heptane containing 1.98 mg mL^−1^ tri-isobutyl aluminum (TiBA, 1.0 M in hexane, Sigma–Aldrich) as a co-catalyst. After filling the system with ethylene (1.2 bar), 18 mg of supported catalyst was added, and polymerization occurred for 1 hour, stirred at 400 rpm.

### Diffusive reflectance infrared Fourier transform (DRIFT) spectroscopy

Diffuse reflectance infrared Fourier transform (DRIFT) spectroscopy data were collected to study the with a Perkin Elmer Frontier instrument, equipped with a Praying MantisTM diffuse reflectance accessory (DRA) and a Mercury–Cadmium–Telluride (MCT) detector. In each experiment, 5–7 mg of the catalyst sample was loaded inside an N_2_-glovebox into a high-temperature reaction chamber (Harrick Scientific Products Inc.) positioned over a porous frit within a nitrogen-filled glovebox. The reaction setup included gas inlets, outlets, and a dome fitted with KBr windows. Before each experiment, all lines were purged with nitrogen. Ethylene polymerization reactions were conducted at room temperature at a 2.5 ml min^−1^ flow rate. The DRIFT spectra were recorded at 30-second intervals in the 4000–600 cm^−1^ range. The *in situ* kinetic curves were obtained by integrating the area of the polyethylene vibrational stretching bands ν(CH_2_)_PE_ and plotted against time. The first derivative of each curve was used to estimate their polymerization rates. The porous and nonporous DRIFTs experiments are performed in triplet using the same catalyst material.

### Scanning electron microscopy

High-resolution scanning electron microscopy (SEM) on the FEI Helios Nanolab G3 operating at 0.1 pA and 2 kV was used to investigate the surface morphology of the MAO-impregnated support and corresponding catalyst. Samples were loaded on Al stubs with carbon tape. The external morphologies were imaged by collecting secondary electrons (SE) with a through-the-lens detector (TLD).

### Fourier transform infrared spectroscopy

To study the Lewis acidity of the MAO-activated chitosan supports and their respective catalysts, Fourier Transform Infrared (FT-IR) spectroscopy and pyridine FT-IR were performed on the Thermo Fischer Nicolet IS5 equipped with a DTGS KBr detector using 32 scans per spectrum from 1000 to 4000 cm^−1^ and 0.482 cm^−1^ resolution.^22^ Samples were prepared inside an N_2_ glovebox using a PIKE Technologies hydraulic press with a force of 1.5 tons. This resulted in pressed pellets (2–5 mg/7 mm diameter) held in position by a stainless-steel collar. The pellets were placed inside a well-sealed IR cell capable of switching between vacuum and probe molecule vapor/gas. No drying treatment was performed since all samples were stored and prepared in an inert and dry atmosphere.

For the pyridine FT-IR spectroscopy measurements, pyridine adsorption occurred for 30 minutes until equilibrium, and spectra were taken every two minutes. After 30 min of evacuation, temperature-programmed desorption (TPD) (5 K min^−1^ ramp to 423 K) under vacuum was applied, and spectra were taken every 10 K. The acidity was quantified using the spectrum, which was taken after 30 min of desorption at 423 K. The temperature was further increased to 823 K to remove all adsorbed pyridine. CO FT-IR spectroscopy measurements were performed on the PerkinElmer 2000 with a MIRTGS detector from 1000 to 4000 cm^−1^ with 0.5 cm^−1^ resolution. CO (10% in He, purity 99.9%) was dosed at 85 K with consequent steps of increasing pressures (between 0.1 mbar and 10 mbar). Spectra were taken after each pulse.1
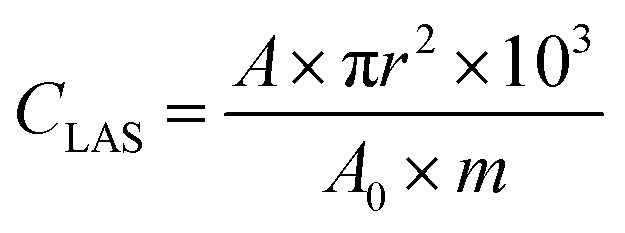


Eqn (1), derived from Beer's law, was used to quantify the concentration of Lewis acid sites *C*_LAS_ (μmol g^−1^) of the samples analyzed with pyridine and CO FT-IR.^34^ A (cm^−1^) represents the integral under the curve delimited by d*δ* (cm^−1^). In the case of pyridine IR spectroscopy, this area was obtained by subtracting the high vacuum IR spectrum from the spectrum taken after 30 min of pyridine desorption at 423 K using the pyridine vibration at 1453 cm^−1^. In the case of CO IR spectroscopy, the area was obtained by subtracting the high vacuum IR spectrum at 85 K from the spectrum at 1 mbar CO pressure using the CO stretching band at 2198 cm^−1^. The apparent integral adsorption coefficient *A*_0_ for the pyridine vibration is 2.22 and 0.95 for the CO vibration.^49^ The effective cross-section *ρ* (mg cm^−2^) is represented by the mass of the pellet *m* (mg) per area of the pellet π*r*^2^ (cm^2^) through which the beam is sent.

### Focused ion beam scanning electron microscopy and energy dispersive X-ray spectroscopy

Focused ion beam scanning electron microscopy (FIB-SEM) images were taken on FEI Helios Nanolab G3 operating at 0.1 nA and 2 kV to investigate the morphology of the synthesized supports and PE particles.^43^ Samples were loaded on Al stubs with carbon tape. The external morphologies were imaged by collecting secondary electrons (SE) with a through-the-lens detector (TLD). Cross-sections of the PE particles were imaged by collecting backscattered electrons (BSE) with the Everhart–Thornley detector (ELD). Before removing half of the catalyst material with Ga FIB under an angle of 52°, a layer of Pt was deposited over the particle *via* FIB-assisted Pt deposition. The exposed cross-sections were cleaned with precision milling. EDX elemental maps of the cross-section were collected with an Oxford instruments Silicon Drift Detector X-Max energy dispersive spectroscope. An acceleration voltage of 15 kV was used to analyze the alumina distribution.

### Inductively coupled plasma-optical emission spectroscopy

The Al (396.153 nm) and Zr (343.823 nm) content of the catalysts were determined by Inductively Coupled Plasma-Optical Emission Spectroscopy (ICP-OES) using a SPECTRO CIROSCCD instrument of SPECTRO Analytical Instruments, after dissolving the solids with aqua regia.

## Conflicts of interest

There are no conflicts to declare.

## Supplementary Material

MA-006-D4MA00893F-s001

## Data Availability

The data supporting this article have been included as part of the ESI.†
