# Supplementary material for: Chitosan microsphere-supported catalysts: design, synthesis and optimization for ethylene polymerization
Source: Mater Adv. 2024 Nov 28;6(1):201–13. doi: 10.1039/d4ma00893f (PMC11604097; doi:10.1039/d4ma00893f)
Supplement: MA-006-D4MA00893F-s001 [file MA-006-D4MA00893F-s001.pdf]

Supplementary Information (SI) for Materials Advances.
This journal is © The Royal Society of Chemistry 2024

COMMUNICATION

Supporting Information

Chitosan Microsphere-supported Catalysts: Design, Synthesis and Optimization for Ethylene Polymerization

Joren M. Dorresteijn*[a], Robin Conradi*[a], Laurens D.B. Mandemaker[a], Kordula Schnabl[a], Virginie Cirriez[b], Alexandre Welle[b], Daniel Curulla-Ferré[b], Florian Meirer[a], Eelco T.C Vogt[a] and Bert M. Weckhuysen[a]

Inorganic Chemistry & Catalysis, Debye Institute for Nanomaterials Science and Institute for Sustainable and Circular Chemistry, Utrecht University, 3584 CG, Utrecht, The Netherlands.

R&D Polymers, TotalEnergies One Tech Belgium, Zone Industrielle C, 7181 Feluy, Belgium

*These authors contributed equally.

Contents

1. Definitive Screening Design (DSD) 2
   Definitive Screening Design 2
  DSD - Experimental Approach 3
   Spray Drying of Chitosan Microspheres for the Definitive Screening Design 3
   DSD - Analysis of Chitosan Microspheres – Effective model selection 4
  DSD - Outcomes and discussion 4
2. Supporting Figures 11
3. References 12

1. Definitive Screening Design (DSD)

The spray dryer's process parameters significantly influence the spray-dried powders' size and morphology.[1] Five different parameters can be controlled. These parameters are concentration of the solution, the inlet temperature of the hot air, the feed rate of the suspension, the spray volume flow, and the aspirator volume flow.

Concentration

The concentration is a measure of the amount of chitosan that has been dissolved in a given amount of acetic acid/water solution.

Inlet temperature

The inlet temperature is the temperature of the heated drying gas. It is the amount of energy provided to dry the solution, and it is directly linked to the wet bulb temperature of the surrounding hot air. When the inlet temperature is lowered, the droplets cannot be dried on time, and some damp precipitate will be stuck inside the drying chamber. A higher inlet temperature is favorable for efficient drying but can also cause product degradation by breaking the chitosan polymer chains.[2] Therefore, the inlet temperature must be high enough to achieve efficient drying but low enough to prevent product degradation.

Feed rate

A peristaltic pump feeds the chitosan solution into the spray nozzle. The feed rate influences how fast the nozzle sprays the solution. If the other parameters stay constant, a higher feed rate increases droplet size because the nozzle automatizes more liquid with the same pressure.[3] Since the droplet size increases, more energy is needed to dry the droplets completely. Otherwise, wet and sticky particles will adhere to the inside of the drying chamber. A higher feed rate should also decrease the outlet temperature because more liquid must be evaporated, which requires more energy. Therefore, a higher feed rate must be compensated for with more efficient drying.

Spray volume flow

Another parameter that significantly influences the particle size is the spray flow. This nitrogen spray flow is injected next to the nozzle, ensuring a cone-like spray of droplets. An increased spray flow disperses the droplets more to the outside of the drying chamber, resulting in smaller droplets. When these droplets dry, the spherical particles will be smaller in size. When the spray flow decreases, larger droplets are created, and a larger particle size should be obtained.[4] However, a lower spray flow prevents optimal atomization and must be sufficiently high to achieve an even droplet size.

Aspirator volume flow

The aspirator volume flow is the volume of hot drying gas that is supplied to the drying chamber. A high drying gas flow rate maximizes the particles' separation efficiency from the air inside the cyclone. In addition, a higher volume flow increases droplet and particle movement inside the drying chamber, which could minimize the air-droplet interaction and the residence time. Therefore, a lower aspirator volume flow should lead to more complete moisture removal.[2]

Definitive Screening Design

All the parameters of the spray dryer are interconnected. If one parameter changes, the other parameters must be altered as well to obtain the desired product. A definitive screening design (DSD) is performed to find the optimal parameters for the spray-dry process to synthesize chitosan with a high enough yield and particle size. A DSD is appropriate for early-stage experimentation to study the influence of different parameters, called factors, on specific variables, called responses. It enables the identification of the factors that have the most substantial effects on a response.[5] The DSD allows for

Please do not adjust margins

studying many factors in relatively few experiments compared to a standard screening design. A minimum run-size DSD can correctly identify main effects with high accuracy. In this case, that DSD can identify with high probability k number of factor-effects is $k < n/2$. In addition, a DSD can also identify any two-factor or quadratic interaction, meaning that two parameters are dependent on each other. Adding experiments, increases the power for the quantification of factor-effects or increases the number of factor-effects than can be identified.

This work uses a definitive screening design to determine the optimal parameters for the efficient production of chitosan microspheres. Using each parameter's lower and upper limits, the statistical software JMP creates a set of experiments that helps establish a reliable model for spray-drying chitosan microspheres. The investigated key performance indicators (KPI’s) are the outlet temperature, yield, and the microsphere's particle size. The spray-dried microspheres were analyzed with SEM to determine size distribution and mean particle size. All results were then analyzed with effective model selection for DSDs methodology.

DSD - Experimental Approach

Spray Drying of Chitosan Microspheres for the Definitive Screening Design

The chitosan microspheres were prepared with a 1, 1.5, and 2 wt.% chitosan (98% purity, ≥75% deacetylated, Sigma-Aldrich) solution in 1 wt% acetic acid (≥99% glacial, ReagentPlus®, Sigma-Aldrich) / water solution. The chitosan was dissolved for 24 hours under stirring at room temperature. About 50-100 ml of the solution was used for each experiment and was spray dried using a BÜCHI B-290 acid-resistant mini spray dryer (Flawil, Switzerland) according to the conditions in Table S2.1. This design of experiments was created by implementing the lower and upper limits of all five continuous factors in the program JMP. The program adds four extra runs to identify any second-order effects. During spray drying, the solution was magnetically stirred at 500 rpm to prevent concentration gradients. The outlet temperature was measured, the dry and white chitosan powder was collected in the collection vessel, and the yield was determined. Additional nozzle cooling was provided by feeding pressurized air into the nozzle to prevent precipitation on the nozzle tip. The standard 1.4 mm in diameter spray nozzle tip and 2.2 mm nozzle cap were used for all experiments.

This journal is © The Royal Society of Chemistry 20xx

Please do not adjust margins

Please do not adjust margins

Table S2.1: *Spray dryer settings of the 1, 1.5, and 2 wt.% chitosan solutions for the definitive screening design experiments.*

Exp #	Aspirator volume flow (m^3/h)	Spray volume flow (L/h)	Feed rate (ml/min)	Inlet temperature (°C)	Concentration (wt.%)
1	31.7	472	15	220	2
2	31.7	357	15	120	2
3	24.1	246	9	120	2
4	21.1	246	15	170	2
5	37.7	246	3	120	2
6	24.1	246	3	220	2
7	37.7	742	3	220	2
8	37.7	246	15	220	1.5
9	24.1	742	3	120	1.5
10	31.7	357	9	170	1.5
11	31.7	246	3	120	1
12	27.1	357	3	220	1
13	37.7	246	9	220	1
14	37.7	742	3	170	1
15	24.1	742	15	220	1
16	37.7	742	15	120	1
17	24.1	246	15	120	1
Opt	37.7	742	3	180	1.5

Table S2.2: *Raw spray dry results per experiment for the definitive screening design and optimized*

Exp #	Outlet Temperature (°C)	Yield (%)	Mean Particle Size (µm)	SD Mean Particle size (µm)	Dominant particle shape
1	142	22.00	9.64	4.96	Satellite
2	88	11.54	9.59	4.07	Wrinkled
3	72	20.00	4.41	2.21	Wrinkled/less satellite
4	102	0.04	13.48	4.57	Satellite - Wrinkled
5	81	0.11	9.12	3.66	Wrinkled
6	123	0.10	12.20	4.28	Satellite
7	140	18.00	5.95	4.19	Satellite
8	140	1.33	9.10	5.93	Satellite
9	74	27.00	3.93	2.02	Wrinkled
10	101	16.67	6.50	3.52	Satellite - Wrinkled
11	83	0.27	3.63	1.58	Rimple/porous
12	135	14.67	5.29	2.39	Satellite
13	138	2.67	4.02	1.84	Mix – satellite
14	114	16.00	4.93	2.64	Wrinkled satellite
15	97	13.33	4.22	2.53	Porous wrinkled
16	58	6.67	4.32	1.76	Porous wrinkled
17	54	0.11	4.56	2.29	Wrinkled
Opt	118	44.02	6.82	3.03	Satellite - Wrinkled

DSD - Analysis of Chitosan Microspheres – Effective model selection

Effective model selection is applied to analyze the results obtained by the definitive screening design. This procedure is tailored explicitly for DSDs, leveraging the structural attributes intrinsic to this experimental design.[6] Effective model selection is a two-stage analysis. In the first stage of this model,

Please do not adjust margins

Please do not adjust margins

every parameter is tested against an estimate, which is constructed from the error variance of the fake factors and center point replicates that are independent of the constructed model. These fake factors are created by adding a fictional extra column in the experimental chart. The estimate is unbiased when assuming no active third or higher-order effects exist. In this approach, every parameter is given a p-value, which is the probability that measures the evidence against the estimate. The p-value of the parameters is tested against a threshold p-value, whose value is dependent on the number of error degrees of freedom. Every KPI has more than two error degrees of freedom, resulting in a threshold p-value of 0.05. When the p-value of the parameter is lower than the threshold p-value, the parameter is considered an active main effect. It thus has a significant influence on the KPIs.

In the second stage of the effective model selection, the second-order effects are estimated from the main effects of the first stage. If there are more than three main effects in the fitting of the definitive screening, half of the second-order effects are reliably identified. If there are three or fewer main factors, all second-order effects are identified reliably. Strong effect heredity is assumed by default, meaning it considers interactions between parameters only if the factors themselves are considered the main effects. It also requires including all lower-order effects. In DSDs, main effects and second-order effects are independent of each other. The second-order effects are also given a p-value according to the same method as the first-order effects to test the significance of the KPIs.

DSD - Outcomes and discussion

The outlet temperature, yield, mean particle size, and particle surface are the most significant key performance indicators (KPIs) to control. The DSD analysis can provide viable feedback on which parameter of the spray-dry process has the most impact on every KPI. The following section will discuss the results obtained from the DSD analysis on these KPIs. The raw results of every DSD analysis experiment can be found in Figure 1 (mean particle size), S1 (outlet temperature) and S2 (yield). The analysis of these results for these KPIs after applying effective model selection can be found in the figures below:

This journal is © The Royal Society of Chemistry 20xx

Please do not adjust margins

Please do not adjust margins

Fit Definitive Screening

Fit Definitive Screening for Outlet Temperature

Stage 1 - Main Effect Estimates

Term	Estimate	Std Error	t Ratio	Prob>|t|
Aspirator volume flow	4,5714	0,6637	6,888	0,0063*
Spray volume flow	-5,286	0,6637	-7,964	0,0041*
Concentration	8,5	0,6637	12,807	0,0010*
Feed rate	-5,786	0,6637	-8,718	0,0032*
Inlet temperature	30,286	0,6637	45,633	<,0001*

Statistic	Value
RMSE	2,4833
DF	3

☑ Quadratic Terms Obey Strong Heredity

☑ Interactions Obey Strong Heredity

Stage 2 - Even Order Effect Estimates

Term	Estimate	Std Error	t Ratio	Prob>|t|
Intercept	100,52	0,8725	115,2	<,0001*
Aspirator volume flow*Concentration	-2,129	0,3833	-5,555	0,0051*
Concentration*Feed rate	9,3633	0,4325	21,648	<,0001*
Concentration*Inlet temperature	2,8633	0,4325	6,6198	0,0027*
Aspirator volume flow*Aspirator volume flow	5,9453	0,9924	5,9911	0,0039*

Statistic	Value
RMSE	1,2605
DF	4

Combined Model Parameter Estimates

Term	Estimate	Std Error	t Ratio	Prob>|t|
Intercept	100,52	1,3044	77,06	<,0001*
Aspirator volume flow	4,5714	0,5036	9,0772	<,0001*
Spray volume flow	-5,286	0,5036	-10,5	<,0001*
Concentration	8,5	0,5036	16,878	<,0001*
Feed rate	-5,786	0,5036	-11,49	<,0001*
Inlet temperature	30,286	0,5036	60,137	<,0001*
Aspirator volume flow*Concentration	-2,129	0,5729	-3,716	0,0075*
Concentration*Feed rate	9,3633	0,6466	14,481	<,0001*
Concentration*Inlet temperature	2,8633	0,6466	4,4282	0,0031*
Aspirator volume flow*Aspirator volume flow	5,9453	1,4835	4,0077	0,0051*

Statistic	Value
RMSE	1,8844
DF	7

Main Effects Residual Plots

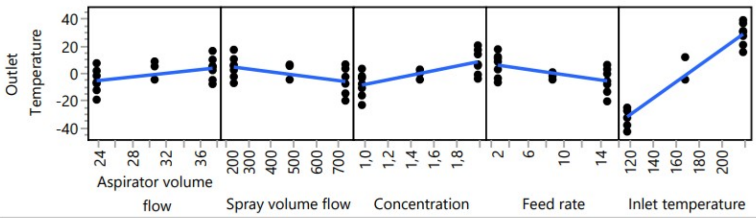

Prediction Profiler

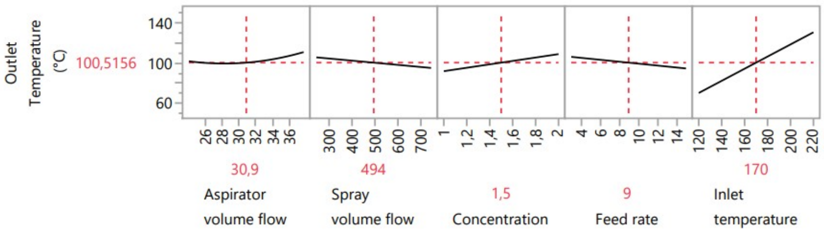

Fit Definitive Screening results of KPI Outlet temperature.

This journal is © The Royal Society of Chemistry 20xx

Please do not adjust margins

Please do not adjust margins

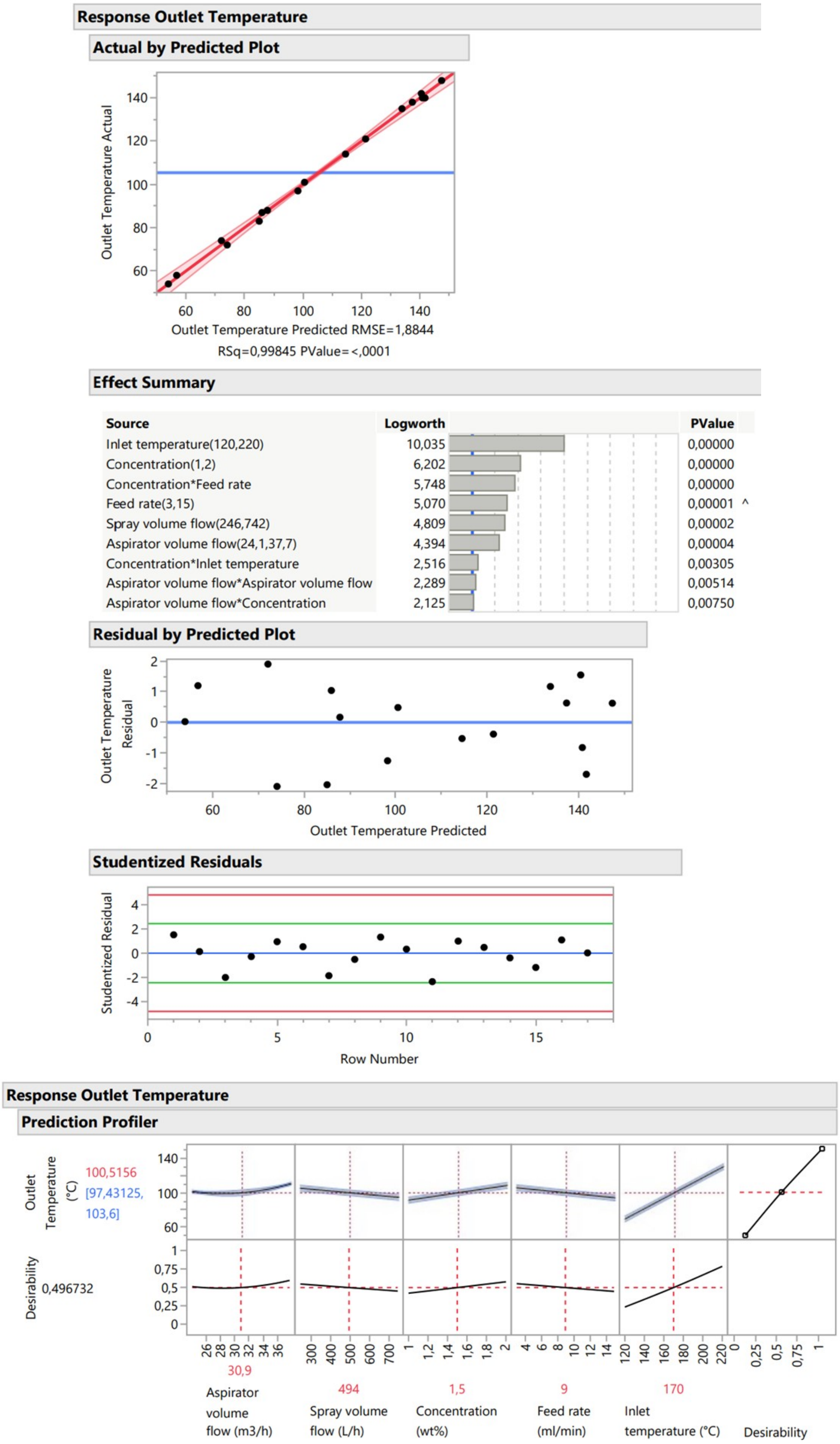

Predicted response of model of KPI Outlet temperature, obtained from fitting least squares of Fit Definitive screening in JMP.

Please do not adjust margins

Please do not adjust margins

Fit Definitive Screening

Fit Definitive Screening for Yield

Stage 1 - Main Effect Estimates

Term	Estimate	Std Error	t Ratio	Prob>|t|
Spray volume flow	8,695	0,7019	12,388	<,0001*
Concentration	1,7093	0,7019	2,4353	0,0590
Feed rate	-1,719	0,7019	-2,45	0,0580

Statistic	Value
RMSE	2,6262
DF	5

☑ Quadratic Terms Obey Strong Heredity

☑ Interactions Obey Strong Heredity

Stage 2 - Even Order Effect Estimates

Term	Estimate	Std Error	t Ratio	Prob>|t|
Intercept	16,84	1,5789	10,666	0,0001*
Spray volume flow*Concentration	2,745	0,6321	4,343	0,0074*
Spray volume flow*Spray volume flow	-4,261	1,419	-3,003	0,0300*
Concentration*Concentration	-3,821	1,419	-2,693	0,0432*

Statistic	Value
RMSE	2,1895
DF	5

Combined Model Parameter Estimates

Term	Estimate	Std Error	t Ratio	Prob>|t|
Intercept	16,84	1,7434	9,6593	<,0001*
Spray volume flow	8,695	0,6462	13,456	<,0001*
Concentration	1,7093	0,6462	2,6453	0,0245*
Feed rate	-1,719	0,6462	-2,661	0,0239*
Spray volume flow*Concentration	2,745	0,6979	3,933	0,0028*
Spray volume flow*Spray volume flow	-4,261	1,5669	-2,719	0,0216*
Concentration*Concentration	-3,821	1,5669	-2,438	0,0349*

Statistic	Value
RMSE	2,4177
DF	10

Main Effects Residual Plots

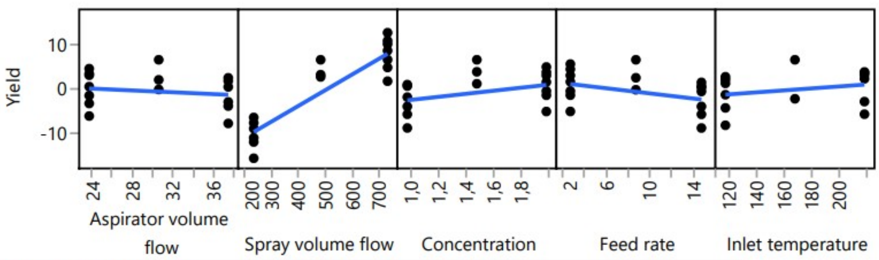

Prediction Profiler

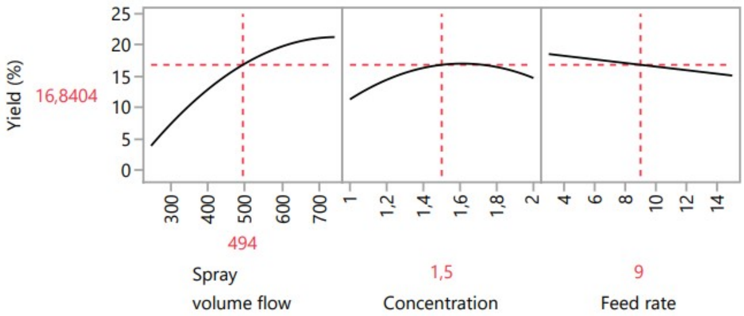

Fit Definitive Screening results of KPI yield in JMP.

This journal is © The Royal Society of Chemistry 20xx

Please do not adjust margins

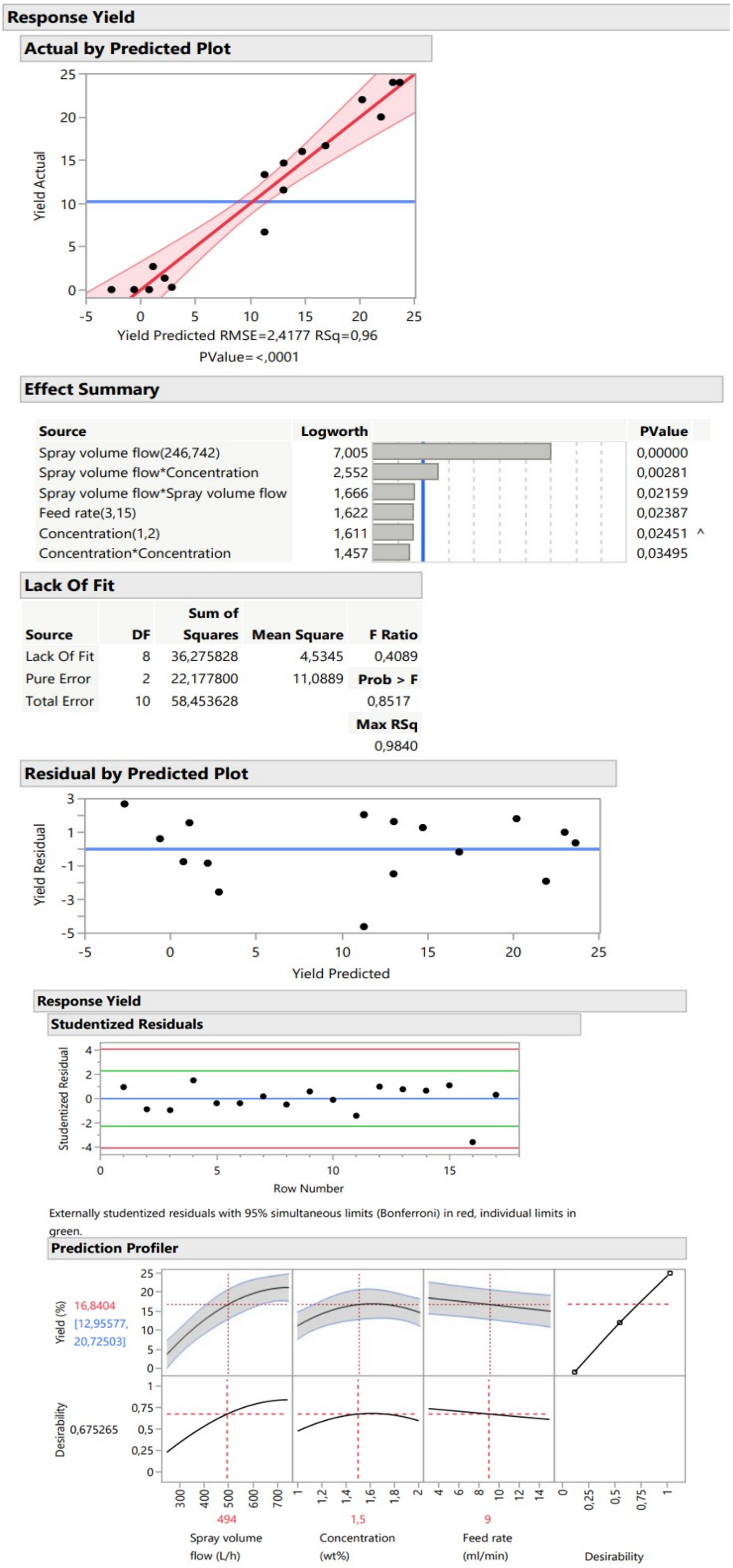

Predicted response of KPI yield model, obtained from fitting least squares of Fit Definitive screening in JMP.

Please do not adjust margins
Please do not adjust margins

Please do not adjust margins

Fit Definitive Screening

Fit Definitive Screening for Mean Particle Size

Stage 1 - Main Effect Estimates

Term	Estimate	Std Error	t Ratio	Prob>|t|
Spray volume flow	-1,338	0,1438	-9,303	0,0007*
Concentration	2,3872	0,1438	16,602	<,0001*
Feed rate	0,7049	0,1438	4,902	0,0080*
Inlet temperature	0,7757	0,1438	5,3948	0,0057*

Statistic	Value
RMSE	0,538
DF	4

☑ Quadratic Terms Obey Strong Heredity
☑ Interactions Obey Strong Heredity

Stage 2 - Even Order Effect Estimates

Term	Estimate	Std Error	t Ratio	Prob>|t|
Intercept	6,7584	0,1092	61,908	<,0001*
Spray volume flow*Concentration	-1,609	0,1345	-11,96	<,0001*
Concentration*Feed rate	0,9048	0,1345	6,7275	0,0011*
Concentration*Inlet temperature	0,7241	0,1345	5,3839	0,0030*

Statistic	Value
RMSE	0,4501
DF	5

Combined Model Parameter Estimates

Term	Estimate	Std Error	t Ratio	Prob>|t|
Intercept	6,7584	0,1191	56,738	<,0001*
Spray volume flow	-1,338	0,1313	-10,19	<,0001*
Concentration	2,3872	0,1313	18,187	<,0001*
Feed rate	0,7049	0,1313	5,37	0,0005*
Inlet temperature	0,7757	0,1313	5,9098	0,0002*
Spray volume flow*Concentration	-1,609	0,1468	-10,96	<,0001*
Concentration*Feed rate	0,9048	0,1468	6,1658	0,0002*
Concentration*Inlet temperature	0,7241	0,1468	4,9343	0,0008*

Statistic	Value
RMSE	0,4911
DF	9

Main Effects Residual Plots

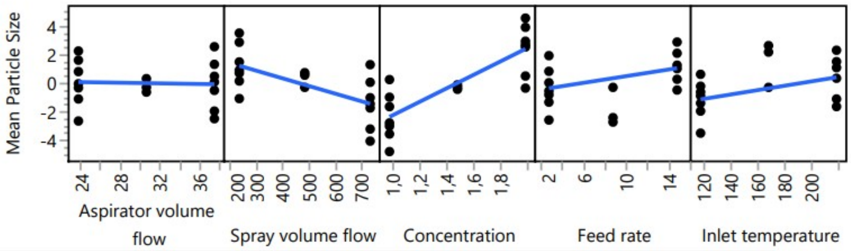

Prediction Profiler

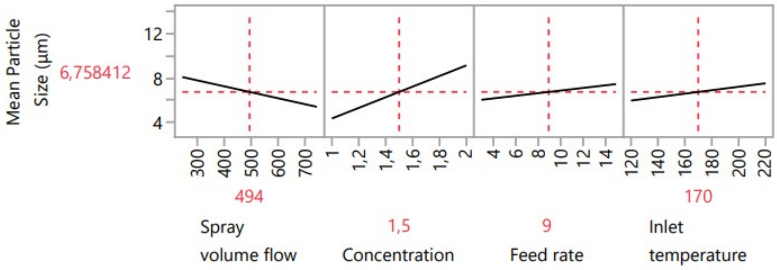

Fit Definitive Screening results of KPI mean particle size in JMP.

Please do not adjust margins

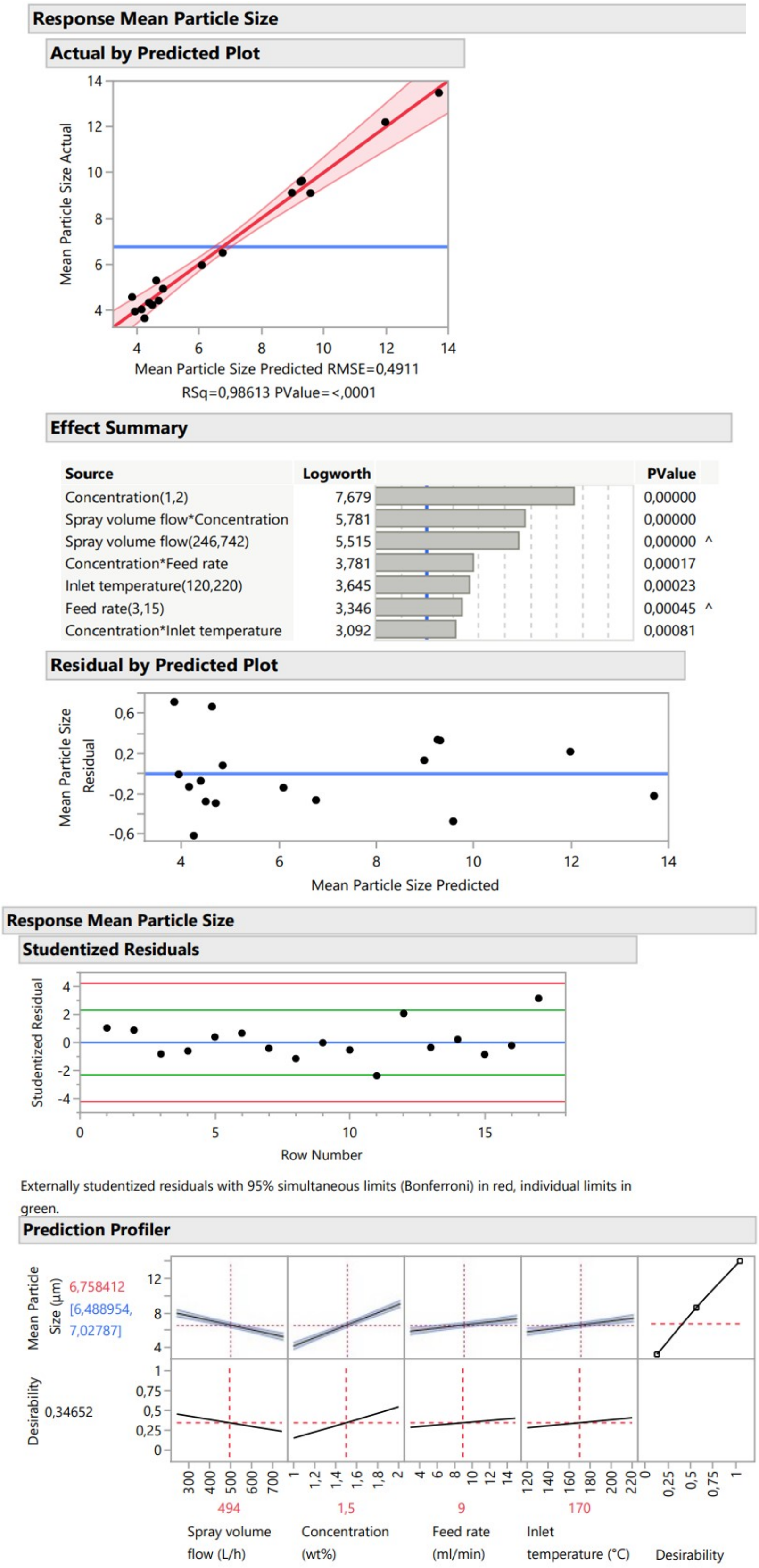

Predicted response of KPI Mean particle size model, obtained from fitting least squares of Fit Definitive screening in JMP.

Please do not adjust margins
Please do not adjust margins

2. Supporting Figures

Parameter	Logworth	P-value
Inlet temperature	10.035	9.23E-11
Concentration	6.202	6.28E-07
Concentration * Feed rate	5.748	1.79E-06
Feed rate	5.070	8.51E-06
Spray volume flow	4.809	0.00002
Aspirator volume flow	4.394	0.00004
Concentration * Inlet temperature	2.516	0.00305
Aspirator volume flow * Aspirator volume flow	2.289	0.00514
Aspirator volume flow * Concentration	2.125	0.00750

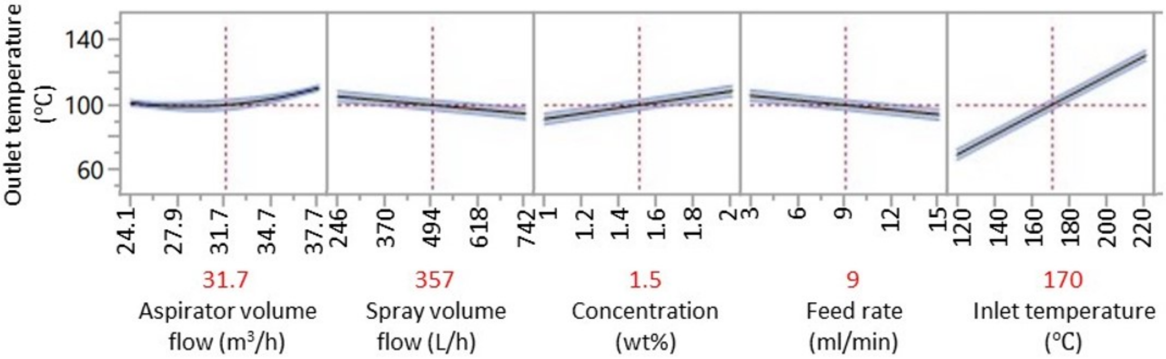

Figure S1: *Top: DSD summary results of performance indicator: Outlet temperature. The gray bar indicates the p-value and logworth for every main and second-order effect. The blue line is the threshold p-value of 0.05. Bottom: Prediction profile extracted from the effective model selection for DSDs representing the dependence of the outlet temperature by the aspirator volume flow, spray volume flow, concentration, feed rate, and inlet temperature. At the minimum required temperature of 100 °C, the optimal value of the parameters is marked in red. The shaded areas represent 95% confidence intervals.*

a)

Parameter	Logworth	P-value
Spray volume flow	7.005	9.89E-08
Spray volume flow * Concentration	2.552	0.00281
Spray volume flow * Spray volume flow	1.666	0.02159
Feed rate	1.622	0.02387
Concentration	1.611	0.02451
Concentration * Concentration	1.457	0.03495

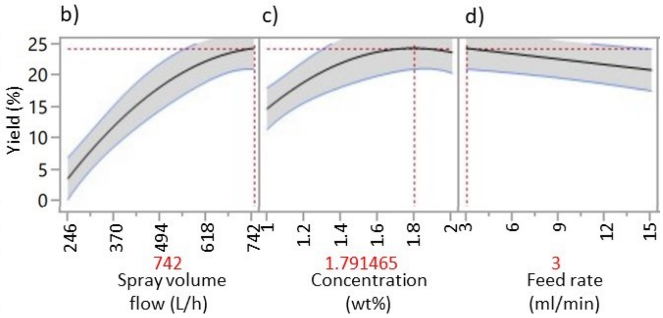

Please do not adjust margins

Figure S2: *(a) DSD summary results of performance indicator: Yield. The p-value and logworth are indicated for every main and second-order effect. The blue line is the threshold p-value of 0.05. (b-d) Prediction profile extracted from the effective model selection for DSDs representing the dependence of the yield by the spray volume flow (b), concentration (c), and feed rate (d). The values of the parameters for a maximized yield are indicated in red. The shaded areas represent 95% confidence intervals*

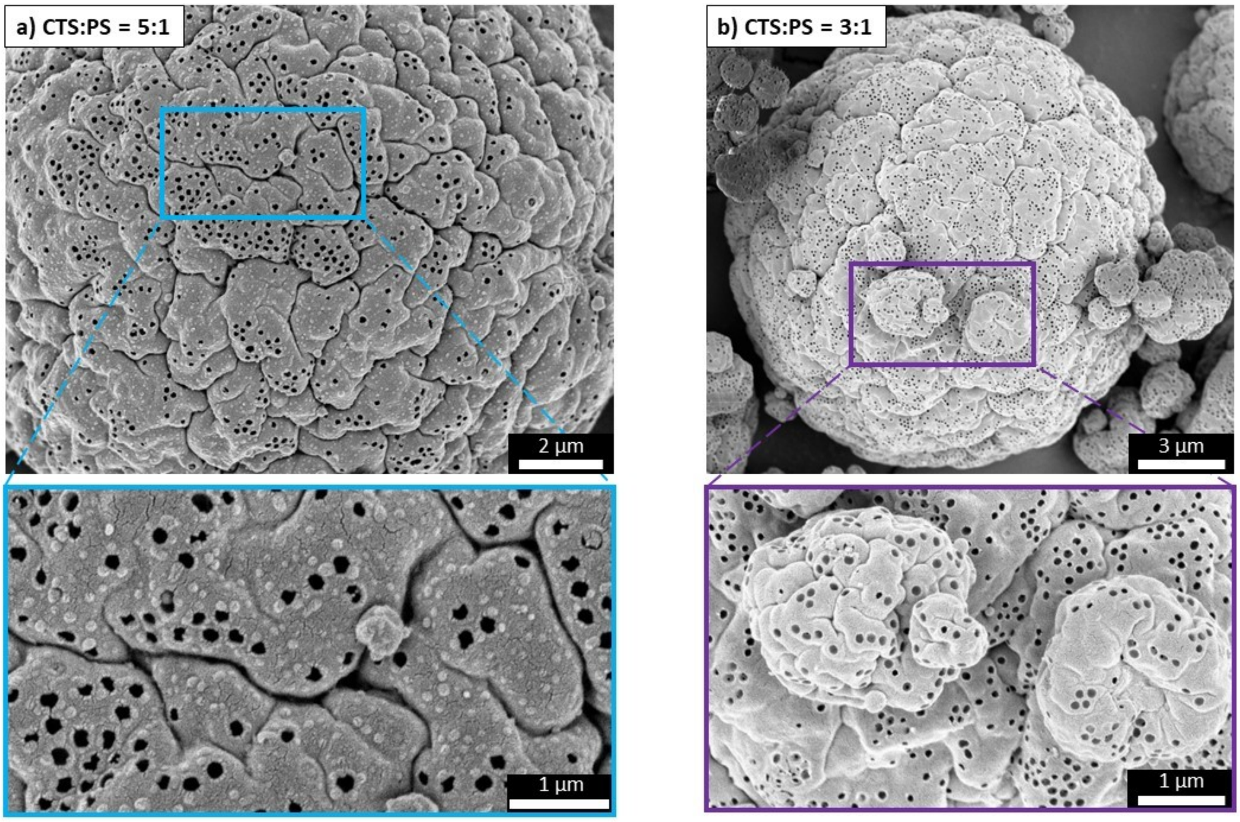

Figure S3: *Porous chitosan microspheres with 150 nm PS spheres with (a) 5:1 and (b) 3:1 chitosan to polystyrene mass ratio.*

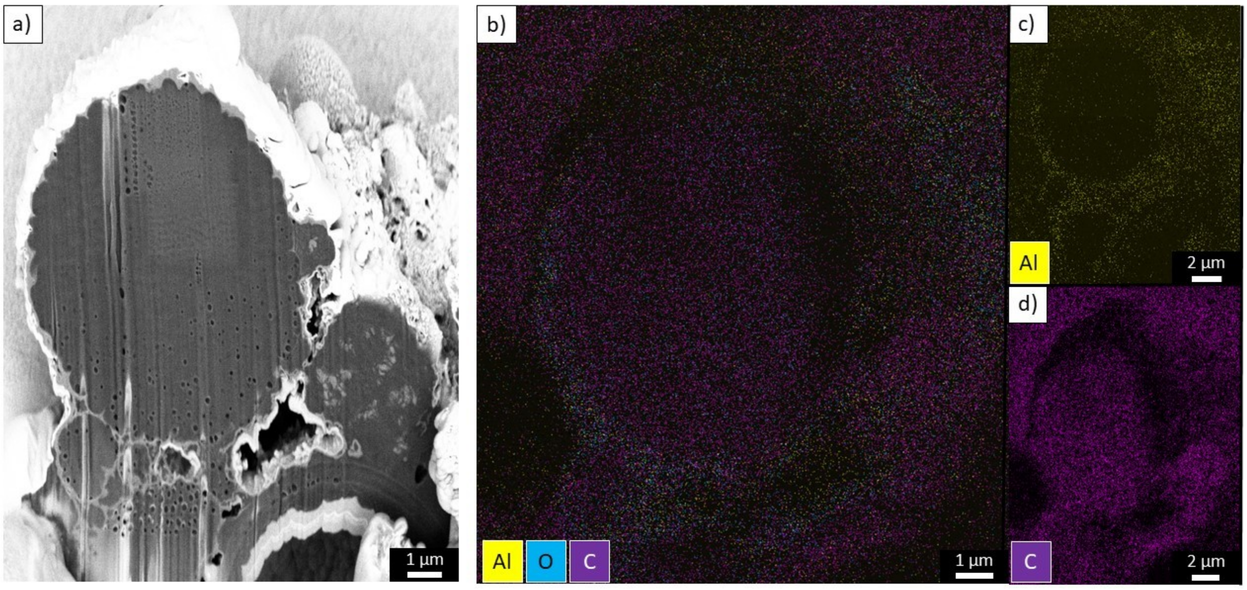

Figure S4: *(a) FIB-SEM cross-section of polyethylene (PE), revealing the fragmentation pattern of the nonporous catalyst after 1-hour of gas phase polymerization. (b) EDX elemental map of the cross-section of the PE particle ca from (a), showing the distribution of aluminum (yellow), oxygen (blue), and carbon (purple) inside the particle. (c) Al K-series of cross-section. (d) C K-series of cross-section.*

This journal is © The Royal Society of Chemistry 20xx

Please do not adjust margins

Please do not adjust margins

3. References

1 P. He, S. S. Davis and L. Illum, Chitosan microspheres prepared by spray dying method, *Eur. J. Pharm. Sci.*, 1996, **4**, S173.

2 D. Santos, A. C. Maurício, V. Sencadas, J. D. Santos, M. H. Fernandes and P. S. Gomes, in *Biomaterials - Physics and Chemistry - New Edition*, InTech, 2018.

3 K. Cal and K. Sollohub, Spray drying technique. I: Hardware and process parameters, *J. Pharm. Sci.*, 2010, **99**, 575–586.

4 X. Li, N. Anton, C. Arpagaus, F. Belleteix and T. F. Vandamme, Nanoparticles by spray drying using innovative new technology: The Büchi Nano Spray Dryer B-90, *J. Control. Release*, 2010, **147**, 304–310.

5 B. Jones and C. J. Nachtsheim, Definitive screening designs with added two-level categorical factors, *J. Qual. Technol.*, 2013, **45**, 121–129.

6 A. Errore, B. Jones, W. Li and C. J. Nachtsheim, Using definitive screening designs to identify active first-and second-order factor effects, *J. Qual. Technol.*, 2017, **49**, 244–264.

This journal is © The Royal Society of Chemistry 20xx

Please do not adjust margins
